# Glucocorticoids induce production of reactive oxygen species/reactive nitrogen species and DNA damage through an iNOS mediated pathway in breast cancer

**DOI:** 10.1186/s13058-017-0823-8

**Published:** 2017-03-24

**Authors:** Renée L. Flaherty, Matthew Owen, Aidan Fagan-Murphy, Haya Intabli, David Healy, Anika Patel, Marcus C. Allen, Bhavik A. Patel, Melanie S. Flint

**Affiliations:** 1 School of Pharmacy and Biomolecular Sciences, Stress, Aging and Disease Group, University of Brighton, Brighton, BN2 4GJ UK; 2 Brighton and Sussex Medical School, Brighton, BN1 9PX UK

**Keywords:** Breast cancer, Stress, Glucocorticoid, Norepinephrine, iNOS

## Abstract

**Background:**

Psychological stress increases the circulating levels of the stress hormones cortisol and norepinephrine (NE). Chronic exposure to elevated stress hormones has been linked to a reduced response to chemotherapy through induction of DNA damage. We hypothesize that stress hormone signalling may induce DNA damage through the production of reactive oxygen species (ROS)/reactive nitrogen species (RNS) and interference in DNA repair processes, promoting tumourigenesis.

**Methods:**

Breast cancer cell lines were incubated with physiological levels of cortisol and NE in the presence and absence of receptor antagonists and inducible nitric oxide synthase (iNOS) inhibitors and DNA damage measured using phosphorylated γ-H2AX. The rate of DNA repair was measured using comet assays and electrochemical sensors were used to detect ROS/RNS in the cell lysates from cells exposed to stress hormones. A syngeneic mouse model was used to assess the presence of iNOS in mammary tumours in stressed versus control animals and expression of iNOS was examined using western blotting and qRT-PCR.

**Results:**

Acute exposure to cortisol and NE significantly increased levels of ROS/RNS and DNA damage and this effect was diminished in the presence of receptor antagonists. Cortisol induced DNA damage and the production of RNS was further attenuated in the presence of an iNOS inhibitor. An increase in the expression of iNOS in response to psychological stress was observed in vivo and in cortisol-treated cells. Inhibition of glucocorticoid receptor-associated Src kinase also produced a decrease in cortisol-induced RNS.

**Conclusion:**

These results demonstrate that glucocorticoids may interact with iNOS in a non-genomic manner to produce damaging levels of RNS, thus allowing an insight into the potential mechanisms by which psychological stress may impact breast cancer.

**Electronic supplementary material:**

The online version of this article (doi:10.1186/s13058-017-0823-8) contains supplementary material, which is available to authorized users.

## Background

Exposure to hormones released as part of the stress response has been linked to an increased risk of diseases such as hypertension, immune dysfunction and cancer [1–3]. There are several genetic and environmental factors that contribute to the formation and metastasis of tumours [4]; however the risks associated with psychological and oxidative stress are yet to be fully explored. The neuroendocrine hormones, glucocorticoids and catecholamines, are able to influence tumour biology through a number of complex systems and are thought to play a role in the initiation and progression of cancer [5]. Glucocorticoids can promote cell survival in breast tumours through glucocorticoid receptor-mediated activation of anti-apoptotic genes [6], and both hormones may alter the immune response, aiding cancer metastases [7].

Stress hormones are now known to play a role in DNA damage and repair, potentially affecting oncogenic transformation [8, 9]. Others have shown that stress hormones can induce DNA damage through the production of reactive oxygen species (ROS) and reactive nitrogen species (RNS) capable of interacting with DNA, causing base changes and strand breaks [10, 11]. Cancers with a propensity to become metastatic have a progressive increase in ROS, contributing to tumour angiogenesis and metastasis [12]. Studies have shown that specific ROS/RNS can sensitise cancer cells to ROS-inducing chemotherapy agents [13, 14]. It is thought that catecholamines have the potential to increase the production of ROS through β2-adrenergic receptor activation, upregulating PKA activation and levels of ROS, generating oxidative phosphorylation within the cell [15]. Additionally, isoforms of the enzyme nitric oxide synthase (NOS), which produce nitric oxide (NO) - a potentially damaging RNS - are upregulated in certain cancers including breast cancer [16]. Overexpression of inducible NOS (iNOS) and the subsequent increase in NO has wide-reaching implications in the context of malignancy, as NO is involved in several central signalling pathways regulating survival and proliferation [17]. Recently the inhibition of iNOS as a potential treatment in breast cancer has been gathering momentum, with studies showing that iNOS inhibition can reduce the growth of tumours [18]. As such this research seeks to draw together direct links between stress hormones and the production of damage inducing ROS/RNS through an iNOS-mediated mechanism in breast cancer.

Functional DNA repair processes are also crucial in order to maintain the genetic integrity of the cell and prevent transformation. Previous work suggests that stress hormones may interact with some DNA repair pathways, and that this interaction in malignant cells slows or halts the rate of repair [19, 20]. In particular, we have shown that the addition of stress hormones allows circumnavigation of DNA damage cell-cycle checkpoints [8], thus, the cell is unable to undergo delay and must replicate with the damaged DNA, increasing the potential of tumourigenic mutations. Furthermore, Hara et al. have shown that the binding of catecholamines to β2-adrenergic receptors recruits the signal transducer proteins β-arrestins. These are able to interfere with the DNA damage response of p53 resulting in down-regulation of normal p53 signalling, another potential mechanism by which deleterious DNA damage is allowed to accumulate [9].

The literature surrounding the field of stress and breast cancer supports the notion that stress signalling has an impact on tumourigenesis; however, the majority of the work is focused on the effects of chronic stress exposure, and the response to acute exposure to stress hormones is not as well-documented. This study aims to test the hypothesis that acute exposure to stress hormones generates increased levels of ROS/RNS and DNA damage, and attenuates DNA repair rates and identifies mechanisms through which this occurs. As previously shown, stress hormones reduce the efficacy of paclitaxel in triple-negative breast cancer (TNBC) through induction of DNA damage [21]. As such, several cell lines were chosen based on their glucocorticoid receptor (GR) expression, in order to investigate the role of ROS/RNS in DNA damage in TNBC versus non-TNBC. The TNBC lines MDA-MB-231 and HCC38 were selected based on their differing GR status, and MCF-7, an estrogen receptor (ER)-positive (ER+) cell line was selected due to its similar level of GR expression to MD-MB-231. These cell lines also possess β2-adrenergic receptors and are aggressively tumourigenic [8]. The breast epithelial cell line MCF10a was also used.

## Methods

### Cells and culture conditions

Breast cancer cell lines MDA-MB-231 and MCF-7 were purchased from ATCC and maintained in Dulbecco’s modified Eagle’s medium (DMEM) (Gibco, UK) with 10% foetal calf serum (Gibco, UK). HCC38 cells were also purchased from ATCC and maintained in Roswell Park Memorial Institute (RPMI) medium (Gibco, UK) with 10% foetal calf serum (Gibco, UK). The 4 T1 breast cancer cell lines were kindly donated by Dr. Hideo Okada (University of California). The 4T1 cells were cultured in DMEM with 4 mM L-glutamine and charcoal-stripped bovine calf serum (10%). MCF10A cells were purchased from ATCC and maintained in HuMEC-ready medium (Thermo Fisher, UK) supplemented with HuMEC supplement kit (Thermo Fisher, UK). All cell lines were maintained in humid conditions at 37 °C and with 5% atmospheric CO_2._ Cell lines were cultured in filtered tissue culture flasks (Fisher, UK) and passaged twice weekly when confluency was reached.

### Hormone treatment

Prior to hormone treatment, cells were seeded in 6-well plates and incubated for 24 h at 37 °C. Cells were treated with predetermined physiologically relevant concentrations of hormones for all experiments unless stated otherwise. The growth medium was removed and replaced with hydrocortisone (Sigma Aldrich, UK) diluted from a stock concentration of 10^-5^ M in medium to a final concentration of 10^-6^ M. Norepinephrine (Sigma Aldrich, UK) was diluted from a stock concentration of 10^-3^ M dissolved in water to a working concentration of 10^-5^ M and then in medium to achieve a final concentration of 10^-6^ M. Pharmacological blocking of hormone receptors to determine specificity was achieved by incubating the cells with the GR antagonist RU486 (Sigma Aldrich, UK) or beta-adrenergic receptor antagonist propranolol (Sigma Aldrich, UK). Antagonists were dissolved first in water and then in medium to achieve a final concentration of 10^-6^ M and cells were incubated in their presence for 30 minutes prior to the addition of cortisol or norepinephrine, respectively.

### Electrochemistry

#### Fabrication and characterisation of ROS/RNS electrodes

Electrodes were fabricated by modification of a previously published approach [22]. Briefly, a conductive composite material made from multiwall carbon nanotubes and epoxy resin was packed into the tip of a plastic pipette tip. The tip was placed flat onto a smooth glass surface and a copper wire was pushed firmly from the end of the plastic tip until it was approximately 2 mm from the end of the plastic pipette tip and the tip was left to set. Following this a glass capillary was inserted over the copper wire and attached to the back of the pipette tip with superglue, attaching the capillary and sealing the electrode. The electrode surface was smoothed, polished and coated in platinum black. The Pt-black composite electrode was then rinsed three times with PBS and then deionised (DI) water before being used. Optimisation and calibration of sensors was achieved by producing a voltamagram measuring a 1 mM ferrocyanide solution. Sensors were characterised using stock solutions and multiple step amperometry was utilised for detection of the various ROS/RNS species. Recordings were carried out in a stirred solution of PBS buffer, where a baseline was achieved and after 10 s a volume of stock solution was added to make the final concentration of the 10 μM of peroxynitrite, DEA-NONOate, hydrogen peroxide and nitrite. For characterisation studies, the current was monitored for hydrogen peroxide (H_2_O_2_), peroxynitrite (ONOO-), NO and nitrite (NO_2_-) at +0.3 V, +0.45 V, +0.62 V and +0.85 V, respectively. The limit of detection for H_2_O_2_ was 3.8 nmol, for ONOO- it was 4 nmol, for NO it was 3.3 nmol and for NO_2_- it was 3.2 nmol.

#### Detection of ROS/RNS from cancer cell lines

MDA-MB-231, MCF-7 and HCC38 cells were plated at a density of 5 × 10^4^ per well and incubated for 24 h. In order to understand the time course during which ROS/RNS generation occurred, cells were exposed to cortisol (1 μM) and NE (1 μM) for 15, 30, and 90 minutes. Control wells were left untreated. Following this period, the medium was removed and cells lysed using 500 μl lysis buffer (Trevigen). Lysates were then collected and ROS/RNS levels were quantified using multiple-step amperometry using a stainless steel counter electrode and non-leak Ag|AgCl reference electrode. Measurements of the current were obtained at +0.3 V, +0.45 V, +0.62 V and +0.85 V for a duration of 30 s.

Additional measurements were also carried out in MDA-MB-231 and MCF-7 cells to understand how ROS/RNS levels were altered when the cells were incubated with RU486 (1 μM) (GR antagonist), propranolol (1 μM) (β-adrenergic receptor antagonist) 1400 W dihydrochloride (10 μM) (iNOS inhibitor; Tocris, UK), L-NAME (100 μM) (non-specific NOS inhibitor; Tocris, UK) and PP2 (10 μM) (Src inhibitor; Abcam, UK) for 30 minutes prior to hormone treatment.

#### Immunofluorescence

For phospho-γ-H2AX analysis, MDA-MB-231 and MCF-7 cells were plated at a density of 2 × 10^5^ per well onto glass coverslips in a 6-well plate and incubated for 24 hrs. They were subsequently exposed to cortisol and NE for 2 h at 37 °C in the presence and absence of RU486 and 1400 W for half an hour prior to hormone treatment; controls were left untreated. For GR localisation, MCF-7 cells were incubated with cortisol in the presence and absence of RU486 and PP2 (10 μM) for 20 minutes. Cells were then fixed in 3% paraformaldehyde 2% sucrose (pH 7.2) PBS for 10 minutes, washed, permeabilised using 0.2% TritonX-100 in PBS for 2.5 minutes at room temperature and blocked with 2% BSA in PBS for 30 minutes at room temperature (RT). Incubation with the primary antibody; anti-phospho-Histone H2AX (1:800 in 2% BSA) (Cell Signalling) occurred for 45 minutes at 37 °C, and anti-GR (1:200 2% BSA) (Insight Biotech, UK) at 4 °C overnight. Samples were incubated with the secondary antibody; anti-mouse/rabbit IgG fluorescein isothiocyanate (FITC) (1:200 in 2% BSA) (Sigma) at 37 °C for 20 minutes. The slides were stained and mounted with Vectashield and visualised using fluorescence microscopy. Fluorescent foci were detected using confocal microscopy (Leica, Germany) and phospho-γ-H2AX-positive cells, categorised as >5 foci, expressed as a percentage of total cells counted.

#### Comet assay

The comet assay was used to measure DNA damage (directly) and DNA repair (indirectly): 1 × 10^6^ cells from the cell lines MDA-MB-231 and MCF10A were isolated and exposed to H_2_O_2_ (50 mM) (Sigma Aldrich, UK), cortisol and NE for 20 minutes. Cells were then either processed immediately or washed in PBS and incubated at 37 °C for a further 20 minutes to allow DNA repair. Cells were then mixed with 1.2% low melting-point agarose (Sigma Aldrich, UK) and pipetted onto slides previously coated with 0.6% ultrapure agarose (Invitrogen, UK). The gels were allowed to set at 4 °C and lysed in comet lysis buffer (Trevigen) before immersion in electrophoresis buffer (50 mM NaOH, 1 mM EDTA, 1% dimethyl sulfoxide (DMSO)) for 45 minutes. Electrophoresis was carried out at 25 V for 25 minutes and the slides were neutralised in 0.4 M Tris pH7. Cells were stained with ethidium bromide and the “comet tails” of 100 cells were scored blind (Nikon, UK) using a 0–4 scoring system based on the length of the tails. Scores are expressed as arbitrary units out of a maximum score of 400.

#### In vivo studies

Tissue processing from the animal work was performed by MF at the University of Pittsburgh, USA; the methodology has been described previously [23]. All mouse protocols were approved by the Institutional Animal Care and Use Committee (IACUC) at the University of Pittsburgh. Briefly, female BALB/c mice (6 weeks old, weight 20 ± 2 g) were injected with 1 × 10^5^ 4 T1 cells/0.2 mL of PBS into the left mammary fat pad. We used the 4 T1 syngeneic mouse model as described previously [24]. The 4 T1 tumours grow at the induction site and metastasise rapidly to the lymph nodes. We selected this model as it is a model of TNBC and is immune competent. The tumours took 2 weeks to become established, with tumour volumes of approximately 100 mm^3^. Tumours were measured twice weekly using a digital calliper and the tumour volumes were calculated using the formula:

$$ \mathrm{V}\mathrm{o}\mathrm{l}\ \left(\mathrm{m}{\mathrm{m}}^3\right) = \mathrm{L} \times {\mathrm{W}}^2/2 $$

in which L is length (mm) and W is width (mm).

Mice were randomised into either a stress group or a no-stress group 3 days before treatment (day -3). At day 0, groups of mice were either placed individually in adequately ventilated tubes for 1 h three times a week (stress) or they experienced no stress (NS). All mice were killed at 4 weeks. All primary tumours were harvested at necropsy. All tumours were histologically confirmed by haematoxylin and eosin (H&E) staining.

### Immunohistochemical assessment

Paraffin-embedded breast tumours were cut into 10-μm-thick transverse sections and stained with H&E. For immunohistochemical assessment, paraffin sections were deparaffinised and rehydrated in serial ethanol. Antigen retrieval was performed with sodium citrate buffer (10 mM, pH6.5) at 95 °C. Samples were incubated with 3% hydrogen peroxide and blocking solution (IHC Select, Millipore) for 1 h. Samples were further incubated with the primary antibody against iNOS (1:200 in PBS/BSA; Thermo Scientific) and subsequently washed with PBS-Tween20 (0.2%). Secondary antibody and streptavidin-horseradish peroxidase (HRP) solutions were applied (IHC Select, Millipore) as per the manufacturer’s instructions, and samples were stained with 3,3-diaminobenzidine (DAB) and counterstained with haematoxylin. Images were obtained and scored blind from 0 − 3, where 0 = no staining, 1 = mild, 2 = moderate and 3 = strong staining.

For immunofluorescence, deparaffinisation and antigen retrieval were performed as above; sections were incubated with 0.1% Triton-X for 5 minutes and blocked with 2% BSA/PBS for 1 h. Sections were incubated with anti-GR (1:50 in PBS/BSA) (Santa Cruz Biotech, UK) for 1 h at RT, washed, and incubated with anti-Rabbit-FITC (1:200) (Sigma Aldrich, UK) for half an hour. Samples were mounted with Vectashield containing 4',6-diamidino-2-phenylindole (DAPI) and imaged using confocal microscopy (Leica, Germany).

### Western blot

MCF-7 cells were plated at a density of 2 × 10^5^ cells per well in a 6-well plate and were incubated with cortisol for 30 minutes or 24 h. Cells were then washed twice with cold PBS and incubated with ice cold radioimmunoprecipitation assay (RIPA) buffer (150 mM NaCl, 1% 10 NP40/Igepal, 0.5% NaDoC, 0.1% SDS, 50 mM protease inhibitor (Sigma Aldrich, UK)) for 1–2 minutes. The lysates were subsequently spun at 13,000 g for 14 minutes at 4 °C and the supernatant was collected to be stored at -20 °C until further use.

A Bradford assay (Sigma Aldrich, UK) was used to measure total protein concentration and 10 μg of protein loaded per sample. Samples were resolved on SDS-PAGE gels (10% resolving and 4.5% stacking) and transferred onto polyvinylidene fluoride (PVDF) membranes. Membranes were blocked in 10% skimmed milk powder (Marvel) and incubated with the following primary antibodies; iNOS 1:2000 in 10% milk (Thermo Scientific, UK) and β-actin 1:3000 (Santa Cruz, USA) overnight at 4 °C. Membranes were subsequently washed in PBS-T (0.2% Tween20) and incubated with appropriate secondary antibodies (Anti-rabbit/mouse, 1:2000, Cell Signalling) in 5% milk for 1 h at room temperature. The membranes were developed using Amersham ECL Prime detection kit, which was prepared as per the manufacturer’s instructions and exposed to Amersham Hyperfilm. The film was then processed using a developing system (Xograph Compact X4) and imaged in a Chemi Imager (Alpha Inotech). Images were analysed using ImageJ software to determine the optical density of the bands.

For immunoprecipitation MCF-7 and MDA-MB-231 cells were incubated with PP2 (10 μM) for 30 minutes prior to 30 minutes with cortisol. Samples were immunoprecipitated for heat shock protein 90 (HSP90) using Dynabeads Protein A precipitation kit as per the manufacturer’s instructions (Thermo Scientific, UK) and anti-HSP90 antibody (Santa Cruz, USA). A Bradford assay (Sigma Aldrich, UK) was used to measure total protein concentration and 10 μg of protein loaded per sample. Membranes were incubated with the primary antibody for SRC 1:2000 in 10% milk (Biosource, UK) and anti-rabbit secondary 1:5000 in 5% milk.

### qRT-PCR

MCF-7 and MCF10A cells were treated with cortisol for 30 minutes and for 24 h (1 μM). For RNA extraction from tumours, 30 mg of tissue per sample was homogenized. RNA was extracted using an RNeasy Kit (Qiagen, UK) and cDNA was synthesised using a Quantitect Reverse Transcription kit (Qiagen, UK) as per the manufacturer’s instructions. A Rotor-Gene SYBR Green (Qiagen, UK) master mix was prepared according to the manufacturer’s instructions using Quantitect Primer Assay for human *NOS2* (Qiagen, UK) or mouse *NOS2*. The sense and antisense primers for mouse *NOS2* were 5′-AATGGCAACATCAGGTCGGCCATCACT-3′ and 5′-GCTGTGTGTCACAGAAGTCTCGAACTC-3′ respectively (Eurofins). cDNA was analysed in the Rotor-Gene qRT-PCR thermocycler and presented as fold change in expression normalised against β-actin.

### Clinical analysis

The expression of *NOS2* and *SRC* in human breast carcinomas was examined using Oncomine Cancer Micorarray database analysis of the The Cancer Genome Atlas (TCGA) Breast database (*n* = 137) and Curtis Breast database, respectively (*n* = 1600). Expression was compared between normal breast tissue and invasive breast carcinoma.

### Statistical analysis

Graphpad Prism v5.0 was used for all statistical analysis. For continuous data assuming normal variance, one-way analysis of variance was used with Tukey’s multiple comparisons test between groups. For discrete data, the Mann-Whitney test was used. Statistical significance was determined when the *p* value was <0.05. All the results are representative of the mean of three independent experiments (*n* = 3), each with three technical replicates ± SEM unless otherwise stated.

## Results

### Exposure to stress hormones increases ROS/RNS production in breast cancer cell lines

**Fig. 1:**
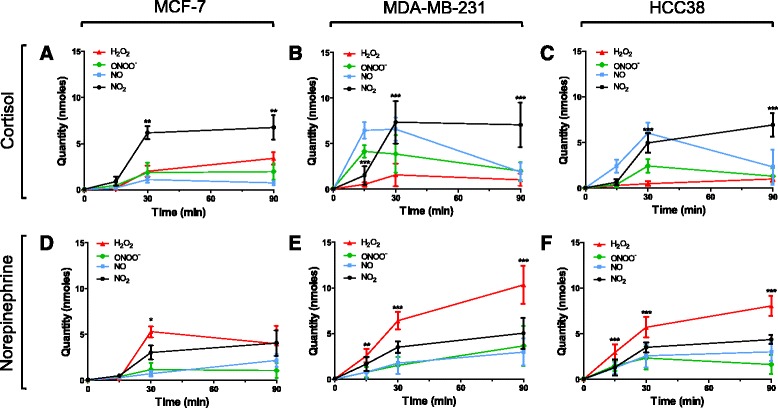
Electrochemical analyses of reactive oxygen species/reactive nitrogen species produced in response to stress hormones over time. MCF-7, MDA-MB-231 and HCC38 cells were incubated with cortisol (1 μM) (**a**-**c**) and norepinephrine (1 μM) (**d**-**f**) for 15, 30 and 90 minutes. Cell lysates were collected and electrochemical were sensors used to measure levels of hydrogen peroxide (*H*_*2*_ *O*_*2*_), peroxynitrite (*OONO* ^*-*^), nitric oxide (*NO*) and nitrogen dioxide (*NO*_*2*_). Data are mean ± SEM and statistical significance was determined using one way analysis of variance (post hoc Tukey multiple comparisons). *Significant increase compared to baseline: *p < 0.05, **p < 0.01, ***p < 0.001

To determine if stress hormones generate ROS/RNS in breast cancer cells, a panel of cell lines (MCF-7, MDA-MB-231 and HCC38) were exposed to physiological concentrations of the stress hormones cortisol and NE (Fig. 1a-f). Electrochemical analysis of cell lysates post hormone treatment revealed that ROS/RNS were generated in a time-dependent manner in all cell lines at 15, 30 and 90 minutes. Treatment with cortisol produced a significant rise in RNS in all cell lines, specifically NO_2_, the stable product of NO and ONOO^-^ generation. Levels peaked significantly at 30 minutes and remained significantly elevated at 90 minutes in MCF-7 (*p* < 0.01) (Fig. 1a), MDA-MB-231 (*p* < 0.001) (Fig. 1b) and HCC38 cells (*p* < 0.001) (Fig. 1c).

After exposure to NE, hydrogen peroxide (H_2_O_2_) levels in MCF-7 cells peaked at 30 minutes (*p* < 0.05) (Fig. 1d). Levels increased immediately and continued to rise significantly for 90 minutes in MDA-MB-231 (*p* < 0.001) (Fig. 1e) and HCC38 cells (*p* < 0.001) (Fig. 1f). Control measurements of untreated MCF-7 and MDA-MB-231 cells were taken at 60 minutes to ensure cell culture practices did not inadvertently generate significant ROS/RNS (Additional file 1: Figure S1A-B).

**Fig. 2:**
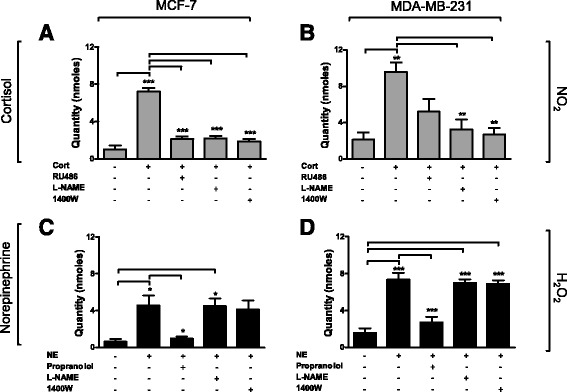
Electrochemical analysis of reactive oxygen species/reactive nitrogen species in response to stress hormones. MCF-7 (**a**) and MDA-MB-231 (**b**) cells were incubated with cortisol (*Cort*) (1 μM) and RU486 (1 μM). MCF-7 (**c**) and MDA-MB-231 (**d**) were also incubated with norepinephrine (*NE*) (1 μM), propranolol (1 μM) and non-specific and specific nitric oxide synthase blockers (NG-nitro-L-arginine methyl ester (*L-NAME*) (100 μM) and 1400 W(10 μM)) for 30 minutes prior to 30 minutes exposure to stress hormone. Levels of hydrogen peroxide (*H*_*2*_ *O*_*2*_) and nitrogen dioxide (*NO*_*2*_) were measured using electrochemical sensors. Data are mean ± SEM and statistical significance was determined using one-way analysis of variance (post hoc Tukey multiple comparisons). *Significant increase compared to unstimulated cells: *p < 0.05, **p < 0.01, ***p < 0.001

MCF-7 and MDA-MB-231 cells were used for further analyses to represent ER+ and TNBC subtypes. The cells were incubated with either the GR antagonist (RU486) or β-AR blocker (propranolol) and either the non-specific NG-nitro-L-arginine methyl ester (L-NAME) or specific (1400 W) NOS inhibitors for 30 minutes prior to hormone exposure. Levels of NO_2_ were significantly increased in MCF-7 (*p* < 0.001) and MDA-MB-231 (*p* < 0.01) cells exposed to cortisol compared to untreated cells (Fig. 2a-b). Levels were decreased in response to incubation with RU486, with a significant reduction seen in MCF-7 (*p* < 0.001) but not in MDA-MB-231 cells compared to cells exposed to cortisol. Furthermore, incubation with L-NAME also blocked the production of NO_2_ in both cell lines compared to cortisol (MCF-7 (*p* < 0.001), MDA-MB-231 (*p* < 0.01)) (Fig. 2a-b) and incubation with 1400 W also blocked NO_2_ generation in MCF-7 cells (*p* < 0.001) (Fig. 2a) and MDA-MB-231 cells (*p* < 0.01) (Fig. 2b). In response to NE, H_2_O_2_ increased significantly in MCF-7 cells (*p* < 0.05) and MDA-MB-231 cells (*p* < 0.001) (Fig. 2c-d) and this effect was negated in the presence of propranolol (MCF-7 (*p* < 0.05), MDA-MB-231 (*p* < 0.001)). Levels of H_2_O_2_ remained unaffected by incubation with NE and L-NAME in MCF-7 cells (*p* < 0.05) (Fig. 2c) and were significantly elevated compared to untreated cells in MDA-MB-231 (*p* < 0.001) (Fig. 2d).

### Stress hormones induce DNA damage in breast cancer cells

**Fig. 3:**
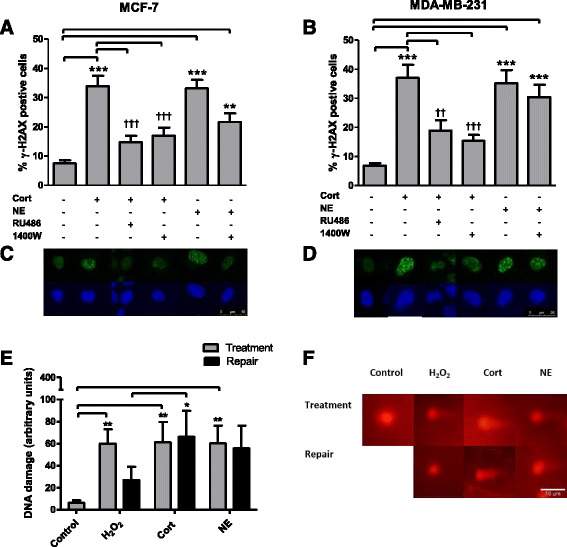
Stress hormones induce DNA damage and impact repair. MCF-7 cells (**a**) and MDA-MB-231 cells (**b**) were incubated with cortisol (*Cort*) (1 μM) and norepinephrine (*NE*) (1 μM) for 2 h in the presence and absence of RU486 (1 μM) and the specific nitric oxide synthase blocker 1400W (10 μM). Cells were immunofluorescently labelled and scored as positive when there were more than five foci. **c**-**d** Representative image of fluorescein isothiocyanate and 4',6-diamidino-2-phenylindole staining in MCF-7 and MDA-MB-231 cells. **e** MDA-MB-231 cells were exposed to cortisol (1 μM) and NE (1 μM) for 30 minutes and assessed for DNA damage using the comet assay. Comet tails indicating DNA strand breaks were visually scored according to intensity (0–4). **f** Representative comet tails in treated and untreated cells and cells after a 20-minute repair period. Data are mean ± SEM and significance was determined using one-way analysis of variance (post hoc Tukey multiple comparisons). *Significant increase compared to the control; ^†^significant decrease compared to cortisol: *p < 0.05, **p < 0.01, ***p < 0.001

Immunofluorescent quantification of the phosphorylation of histone H2AX was used as an indicator of DNA damage in breast cancer cells treated with stress hormones. MDA-MB-231 and MCF-7 cells were incubated with cortisol and NE at physiologically relevant concentrations for 2 h, and with RU486 and the selective iNOS inhibitor 1400 W. Analysis of the number of cells containing more than 5 fluorescent foci, expressed as a percentage of total cells counted, indicated a statistically significant increase in DNA damage in response to cortisol in MCF-7 (33%) (*p* < 0.001) (Fig. 3a) and MDA-MB-231(37%) (*p* < 0.001) (Fig. 3b) cells compared to untreated cells (7.5% and 6.8%, respectively).

The addition of NE also produced a significant increase (*p* < 0.001) in DNA damage of 33% in MCF-7 (*p* < 0.001) and 35% in MDA-MB-231 (*p* < 0.001) cells compared to untreated cells (Fig. 3a-b). Furthermore, in both cell lines the effect of cortisol was nullified in the presence of RU486, indicating this effect was receptor-mediated (14% and 18%; *p* < 0.01). Incubation with the iNOS inhibitor prior to cortisol treatment reduced the levels of DNA damage by greater that 50% in MCF-7 (*p* < 0.001) and MDA-MB-231 (*p* < 0.001) cells, but had no significant effect on NE-treated cells, suggesting the cortisol but not NE acts through iNOS to produce damaging RNS. Representative phospho-γ-H2AX images for MCF-7 (Fig. 3c) and MDA-MB-231 (Fig. 3d) cells are shown. MCF10A cells were also exposed to cortisol and NE and DNA damage was measured using the comet assay (Additional file 2: Figure S2). There was no significant increase in DNA damage in response to cortisol or NE.

### DNA repair is adversely affected after stress hormone exposure

To understand if stress hormone exposure affects the ability of the cell to repair DNA we used the comet assay. DNA repair was monitored by incubating cells after treatment with stress hormones and measuring the damage remaining after 20 minutes. MDA-MB-231 cells were exposed to cortisol and NE for 30 minutes and DNA damage was assessed; a separate plate of cells was exposed to cortisol and NE for 30 minutes and incubated for a further 20 minutes in medium alone to allow DNA repair. Comet tails indicating DNA strand breaks were visually scored according to intensity (0–4). Similar to the γ-H2AX assay, a significant increase in DNA damage was immediately observed compared to the control after 30 minutes treatment with cortisol and NE (*p* < 0.01) (Fig. 3e). Damage was also observed in the H_2_O_2_ control (*p* < 0.01), and this was reduced after a 20-minute repair period. However, the levels of DNA damage in the cells that was allowed to repair post cortisol treatment remained significant compared to the H_2_O_2_ DNA repair control (*p* < 0.05), indicating less DNA repair. Representative images are shown in Fig. 3f.

### iNOS expression is upregulated in invasive breast carcinoma and mammary tumours in mice exposed to stress

**Fig. 4:**
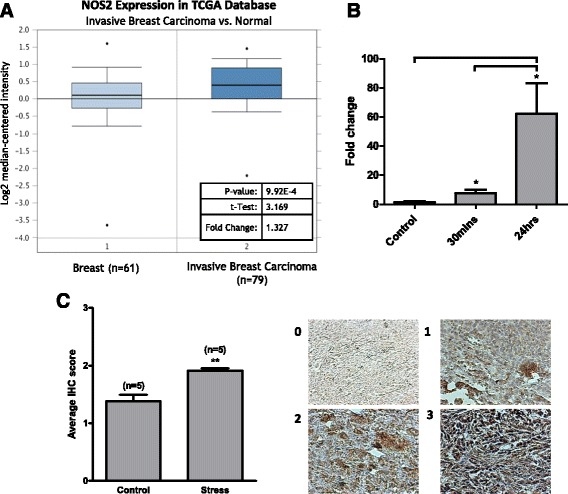
Expression of inducible nitric oxide synthase (iNOS) is increased in breast carcinoma and mouse mammary tumours in response to stress. **a** Oncomine Cancer Mircoarray databases were used to analyse expression of *NOS2* in the The Cancer Genome Atlas (*TGCA*) Breast database (*n* = 137). Expression was compared between normal breast tissue (*n* = 61) and invasive breast carcinoma (*n* = 79). **b** MCF-7 cells were exposed to cortisol (1 μM) for 30 minutes and 24 h and mRNA was extracted. cDNA was synthesised and amplified in the presence of gene-specific primers for *NOS2* and β-actin using qRT-PCR. Cycle threshold (Ct) values for *NOS2* were normalised against β-actin and fold change was calculated using the delta-Ct method. Data are mean ± SEM and significance was determined using one-way analysis of variance (post hoc Tukey multiple comparisons). **c** The 4T1 mouse mammary gland cells were transplanted into the fourth mammary fat pad of female BALB/C mice and the animals were randomised into groups exposed to either acute restrain stress or no stress. Tumours were harvested, fixed in paraffin and sectioned subsequent to immunohistochemical detection (*IHC*) of iNOS. Labelling was scored (0–3) according to intensity; representative panels are shown. The Mann-Whitney test was used to ascertain statistical significance. *Significant increase: *p < 0.05, **p < 0.01, ***p < 0.001

Expression of the gene encoding iNOS (*NOS2*) was analysed using Oncomine Cancer Microarray databases. In invasive breast carcinoma (n = 53) *NOS2* expression was significantly increased (*p* = 7.19E-9) compared to normal stromal breast tissue (n = 6) (Fig. 4a). To examine if iNOS expression was regulated at the gene level, mRNA was extracted from MCF-7 cells exposed to cortisol for either 30 minutes or 24 h. A significant increase in levels of iNOS mRNA was observed at 24 h compared to the 30-minute time point and the control (*p* < 0.05) (Fig. 4b). Western blot analysis of protein extracted from MCF-7 cells indicated no increase in iNOS after 30 minutes exposure to cortisol. However after 24 h exposure there was a trend towards increased expression (*p* = 0.15) (Additional file 3: Figure S3).

Immunohistochemical assessment (IHC) was used to determine the iNOS protein levels in tumours isolated from mice injected with 4T1 cells and either exposed to acute psychological stress or no stress. Paraffin-embedded sections were incubated with a primary antibody against iNOS and the intensity of staining was scored from 0–3, where 0 = 0% staining, 1 = ≤15%, 2 = 15–50% and 3 = 50–100%. Both the non-stressed (NS) and stressed groups scored positively for iNOS within the tumours; however, samples from psychologically stressed animals displayed a statistically significant (*p* < 0.001) higher average IHC score than NS mice (Fig. 4c). The 4T1 tumours from NS and stressed mice were also positively stained for cytoplasmic and nuclear GR (Additional file 4: Figure S4). Tumour weight and volume has been previously published [23]. There was no significant difference in primary tumour weight or volume between the NS and acutely stressed groups, which is typical for restraint stress studies [25].

### Glucocorticoid-induced production of RNS is reduced by Src kinase inhibition

**Fig. 5:**
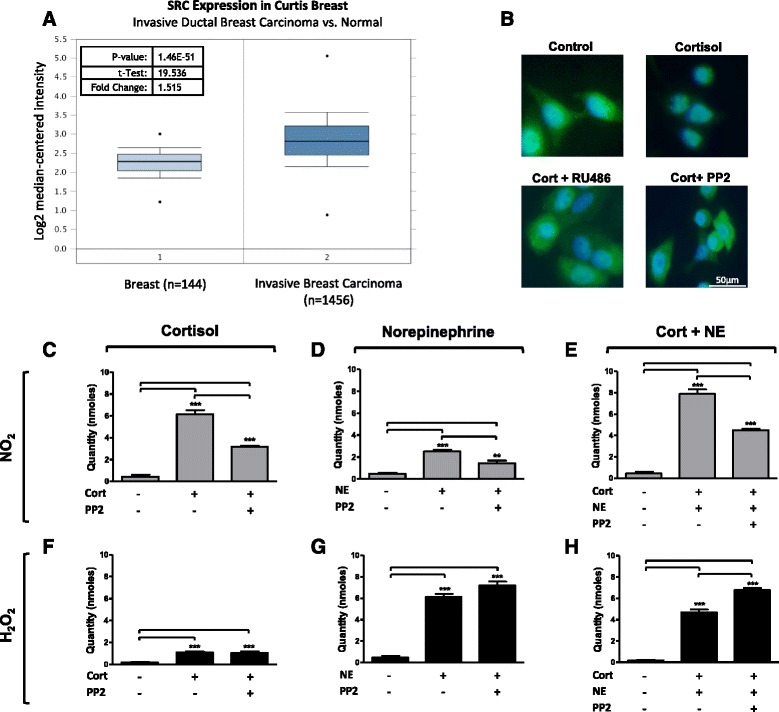
Src expression is increased in breast carcinoma and inhibition of Src blocks cortisol-induced reactive nitrogen species. **a** Oncomine Cancer Microarray databases were used to analyse expression of *SRC* in the Curtis Breast database (n = 1600). Expression was compared between normal breast tissue (n = 144) and invasive breast carcinoma (n = 1456) (*p* = 1.46E-51). **b** MCF-7 cells were exposed to cortisol (*Cort*) (1 μM) for 20 minutes and to the glucocorticoid receptor (GR)-antagonist RU486 (1 μM) and Src inhibitor PP2 (10 μM) for 30 minutes. Cells were immunofluorescently labelled for the GR and counterstained with 4',6-diamidino-2-phenylindole. Representative images are shown. MCF-7 cells were incubated with cortisol (1 μM) (**c**, **f**), norepinephrine (*NE*) (1 μM) (**d**, **g**) or a combination of both (**e**, **h**) for 30 minutes after 30 minutes exposure to PP2 (10 μM). Levels of hydrogen peroxide (*H*_*2*_ *O*_*2*_) and nitrogen dioxide (*NO*_*2*_) were measured using electrochemical sensors. Results were analysed using analysis of variance (post hoc Tukey multiple comparisons). Data are mean ± SEM: *p < 0.05, **p < 0.01, ***p < 0.001

A multi-complex of proteins including heat shock proteins and Src kinase is associated with the GR in its ligand-unbound state [26, 27]. Expression of Src was analysed using Oncomine Cancer Microarray databases. In invasive breast carcinoma (Curtis Breast database, *n* = 1456) *Src* expression was significantly increased (*p* = 1.46^-51) compared to normal breast tissue (*n* = 144) (Fig. 5a). MCF-7 cells were incubated with cortisol alongside the Src inhibitor PP2 and GR-antagonist RU486. Immunofluorescent analysis shows cortisol induced translocation of the GR to the nucleus, and this was blocked by incubation with RU486 and PP2 (Fig. 5b). Western blot analysis of cells treated with cortisol and PP2 and immunoprecipitation for HSP90 confirmed presence of the Src-HSP90 complex (Additional file 5: Figure S5).

To determine if GR-associated Src is involved in the generation of RNS, electrochemical sensors were used to detect ROS/RNS in cell lysates in MCF-7 cells. A significant decrease in NO_2_ was observed in response to cortisol and Src inhibitor PP2 compared to cortisol alone (*p* < 0.001) (Fig. 5c). Similarly a significant decrease in NO_2_ was also observed in response to NE and PP2 compared to NE alone (*p* < 0.01) (Fig. 5d). Cortisol and NE in combination have an additive effect on the generation of NO_2_, increasing it significantly compared to the control (*p* < 0.001), and this effect was reduced by PP2 (*p* < 0.001) (Fig. 5e). H_2_O_2_ increases in response to cortisol and remained elevated with the addition of PP2 (Fig. 5f). NE-mediated levels of H_2_O_2_ were unaffected by the addition of PP2 with increases compared to the control (*p* < 0.001) (Fig. 5g). Cells treated with cortisol and NE in combination produced higher levels of H_2_O_2_ when incubated with PP2 than when treated with cortisol or NE alone (*p* < 0.001) (Fig. 5h).

## Discussion

Our results show that acute exposure to stress hormones can induce DNA damage and that the efficacy of the subsequent repair is also affected. Using electrochemical methods a real-time increase in ROS/RNS was observed as a result of either cortisol or NE incubation. The ability of chronic stress hormones exposure to induce DNA damage in breast cancer has recently been shown by our group [21]. The results corroborate our assertion that both catecholamines and glucocorticoids, at physiological levels, can increase DNA damage through receptor-mediated signalling. The use of electrochemical analyses to quantify production of ROS/RNS in response to ferrocifens in breast cancer has also been explored previously [28], indicating that TNBC cells produce ROS/RNS in response to stimuli.

Our main findings indicate that the effect of cortisol on both the production of RNS and DNA damage is abrogated in the presence of NOS and iNOS inhibitors (L-NAME and 1400 W). This not only suggests that glucocorticoids induce ROS-mediated DNA damage, but that they also have a previously unobserved effect on the activity of iNOS. This is a particularly relevant finding in breast cancer as the expression of iNOS has been found to increase in line with tumour grade and progression [29], indicating that NO activity may drive malignant growth under certain circumstances [18, 30, 31]. As such, NOS inhibitors are emerging as an area of investigation for potential treatments to combat the tumorigenic effects of NO [32]. However, the role of NO in the pathophysiology of cancer is complex, with low levels mediating many homeostatic processes and allowing cell proliferation, while high levels are associated with cytotoxicity and can induce apoptosis [33].

It is unclear as to whether or not the rise in NO in response to stress hormones is a protective mechanism, or whether it is as a result of increased cellular activity caused by DNA damage at this time. However, it is possible to conclude that the generation of RNS must in part be influenced by the GR, as the presence of a receptor antagonist reduces both the DNA damage and production of RNS. Within the cells lines there is some variation in the effect of the GR antagonist RU486, with a more prominent reduction seen in MCF-7 cells. This may be attributed to the cell line MCF-7 expressing a higher percentage of total GRs compared to MDA-MB-231 cells [21], and to the inhibitory effect of RU484 on progesterone receptors, also present on MCF-7 but not MDA-MB-231 cells [34].

The expression of iNOS protein was found to increase in mammary tumours from mice experiencing chronic psychological stress compared to non-stressed controls. In breast cancer cell lines although iNOS mRNA expression was increased after 24 h exposure to cortisol, protein expression remained unchanged. The actions of glucocorticoids have classically been described as genomic, mediated through the GR; however, glucocorticoids have been shown to induce almost immediate non-genomic actions on other signalling processes as a result of proteins dissociating from the GR complex [27, 35, 36]. The complex includes proteins such as HSP90 and Src, a multifunctional protein involved in survival, proliferation and angiogenesis [37]. Src kinases are overexpressed in many cancers including breast cancer and can be used as metastatic markers [38, 39]. The activation of Src via phosphorylation as a result of downstream adrenergic signalling has also been identified as a key switch in tumour metastases, with Src implicated in NE-mediated vascular endothelial growth factor (VEGF) and IL-6 production, ultimately promoting invasion and metastases [40]. Furthermore, Src is capable of phosphorylating iNOS in breast cancer cells, prolonging their half-life and promoting NO generation [41].

**Fig. 6:**
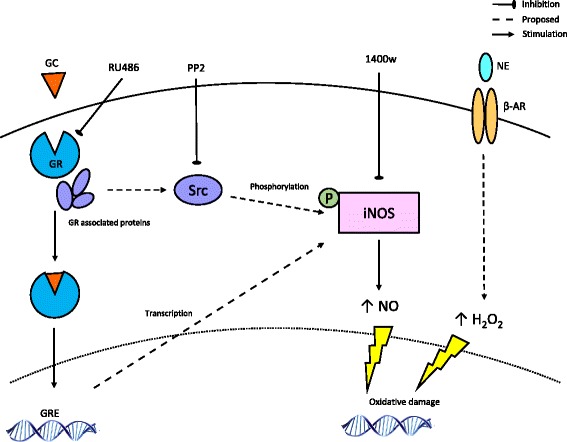
Potential pathway through which stress hormones may stimulate the production of reactive nitrogen species (RNS)/reactive oxygen species (ROS). Glucocorticoid (GC) and norepinephrine (NE) induce DNA damage through the production of RNS/ROS. Activation of the glucocorticoid receptor (GR) may facilitate non-genomic effects on inducible nitric oxide synthase (iNOS) through a post-translational modification. Binding of glucocorticoids to the GR promotes translocation to the nucleus to act on GR response elements (*GRE*), facilitating transactivation or transrepression of genes. Binding also induces conformational changes including dissociation of a multiprotein complex comprising heat shock proteins (HSP) and Src. Src may then mediate phosphorylation of iNOS extending its half-life and promoting the generation of nitric oxide (NO). *H*_*2*_ *O*_*2*_ hydrogen peroxide, *β-AR* β-Adrenergic receptor

In this study inhibition of Src using PP2 attenuated the glucocorticoid induced production of RNS; however, it should be noted that PP2 has also been found to inhibit other members of the Src family of protein kinases [42]. As such our data may suggest a potential mechanism through which glucocorticoid binding to the GR may indirectly exert a non-genomic effect on iNOS to produce damaging levels of RNS, a previously unexplored action of glucocorticoids (Fig. 6).

Interestingly, DNA damage was not significantly reduced in cells exposed to the selective iNOS inhibitor prior to treatment with NE, indicating that, unlike cortisol, NE does not interact with or induce iNOS. Moreover the composition of levels of ROS/RNS produced by NE-treated samples differed greatly from that of the samples treated with cortisol. In NE-treated cells a significant increase was seen in the generation of H_2_O_2_ compared to other ROS/RNS, and this effect was reversed through inhibition of the β-adrenergic receptor using propranolol. The mechanism through which the β-adrenergic receptor induces the generation of ROS is still unclear; however, it is thought that this may be as a result of Gs-PKA signalling [9]. Incubation with both hormones produces the biggest effect on the production of RNS (Fig. 5f), indicating that the mechanisms of DNA damage and ROS/RNS generation in these two stress hormones are distinct from each other; however, in combination they may have an additive effect.

## Conclusion

The effects of acute exposure to stress hormones can be seen to have a significant impact on breast cancer cells, increasing intracellular levels of ROS/RNS and DNA damage and negatively affecting repair processes. These results also demonstrate that glucocorticoid receptor-mediated signalling may indirectly interact with iNOS to produce these damaging levels of RNS. In addition, synthetic glucocorticoids such as dexamethasone are regularly prescribed alongside conventional chemotherapy for hypersensitivity. With this in mind, the work presented here provides an important understanding of how endogenous and exogenous glucocorticoids can regulate ROS/RNS, which may impact response to current and emerging treatments. In summary, these data provide an insight into the potential mechanisms by which psychological stress, glucocorticoid receptor signalling and iNOS activity may influence the progression and treatment of breast cancer.

## Additional files

Additional file 1: Figure S1. ROS/RNS detection controls. Untreated MCF-7 (**a**) and MDA-MB-231 (**b**) were incubated alongside treatment wells and lysed at 0 and 60 minutes. Cell lysates were collected and electrochemical sensors used to measure levels of hydrogen peroxide (*H*_*2*_ *O*_*2*_) and nitrogen dioxide (*NO*_*2*_). (PPTX 111 kb)

Additional file 2: Figure S2. Stress hormones do not induce DNA damage or iNOS expression in a non-tumourigenic mammary epithelial cell line. **a** MCF10A cells were exposed to cortisol (1 μM) and NE (1 μM) for 30 minutes and assessed for DNA damage using the Comet assay. Comet tails indicating DNA strand breaks were visually scored according to intensity (0–4). Representative images shown. **b** MCF10A cells were exposed to cortisol (1 μM) for 30 minutes and 24 h and mRNA extracted. cDNA was synthesised and amplified in the presence of gene specific primers for *NOS2* and β-actin using qRT-PCR. Ct values for *NOS2* were normalised against β-actin and fold change calculated using the delta-Ct method. Mean ± SEM is expressed and significance was determined using one-way ANOVA (post hoc Tukey multiple comparisons); *significant increase, *p < 0.05, **p < 0.01, ***p < 0.001. Technical replicate (*n* = 3). (PPTX 125 kb)

Additional file 3: Figure S3. Expression of iNOS protein is unchanged in response to cortisol. MCF-7 cells were exposed to cortisol (1 μM) for 30 minutes or 24 h. iNOS protein expression was visualised using western blotting. Optical density values were normalised against β-actin. Mean ± SEM is shown. (PPTX 186 kb)

Additional file 4: Figure S4. Glucocorticoid receptor localisation in mice mammary tumours. The 4T1 mouse mammary gland cells were transplanted into the fourth mammary fat pad of female BALB/C mice and the animals randomised into groups either exposed to acute restraint stress or no stress. Tumours were harvested, fixed in paraffin and sectioned subsequent to immunofluorescent detection of glucocorticoid receptor (*GR*). Representative panels are shown. (PPTX 414 kb)

Additional file 5: Figure S5. Cortisol induces the dissociation of Src from HSP90. MCF-7 and MDA-MB-231 cells were exposed to cortisol (1 μM) for 30 minutes alongside PP2 (10 μM). Cell lysates were immunoprecipitated for HSP90 and protein levels of Src were visualised using western blotting. (PPTX 52 kb)

## Abbreviations

ANOVA: analysis of variance
β-AR: β-Adrenergic receptor
BSA: Bovine serum albumin
Cort: Cortisol
ER: Estrogen receptor
FITC: Fluorescein isothiocyanate
GR: Glucocorticoid receptor
H&E: Haematoxylin and eosin
IHC: Immunohistochemical assessment
iNOS: Inducible nitric oxide synthase
L-NAME: NG-nitro-L-arginine methyl ester
NE: Norepinephrine
NO: Nitric oxide
PBS: Phosphate-buffered saline
ROS: Reactive oxygen species
RNS: Reactive nitrogen species
RT: Room temperature
TNBC: Triple-negative breast cancer
