# Supplementary figures and images for: Glucocorticoids induce production of reactive oxygen species/reactive nitrogen species and DNA damage through an iNOS mediated pathway in breast cancer
Source: Breast Cancer Res. 2017 Mar 24;19:35. doi: 10.1186/s13058-017-0823-8 (PMC5366114; doi:10.1186/s13058-017-0823-8)

## Slide 1
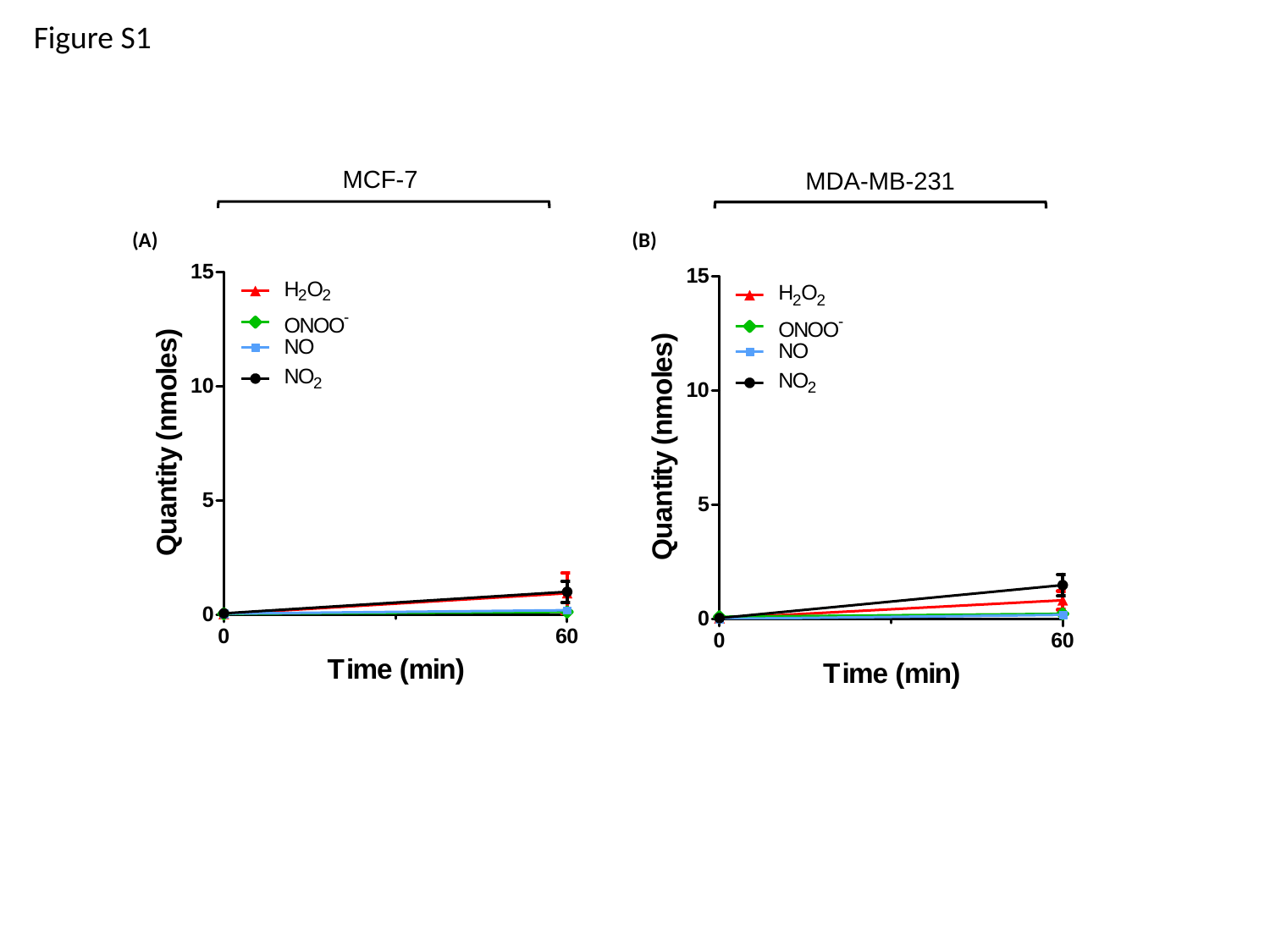

Figure S1
MCF-7
MDA-MB-231
(B)
(A)

Supplement: Supplementary file 1 — ROS/RNS detection controls. Untreated MCF-7 (a) and MDA-MB-231 (b) were incubated alongside treatment wells and lysed at 0 and 60 minutes. Cell lysates were collected and electrochemical sensors used to measure levels of hydrogen peroxide (H 2 O 2) and nitrogen dioxide (NO 2). (PPTX 111 kb) [file 13058_2017_823_MOESM1_ESM.pptx]

## Slide 1
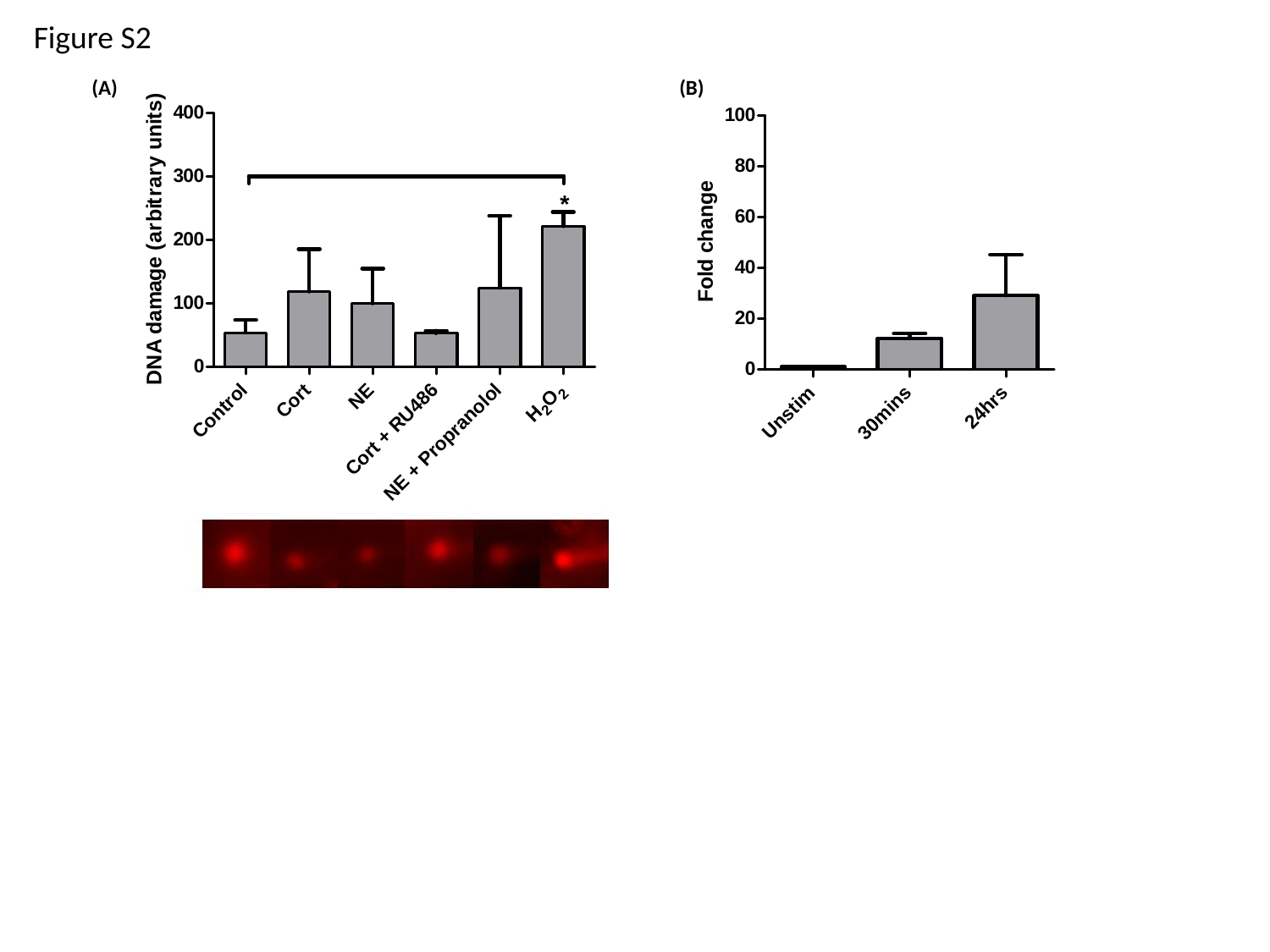

Figure S2
(B)
(A)

Supplement: Supplementary file 2 — Stress hormones do not induce DNA damage or iNOS expression in a non-tumourigenic mammary epithelial cell line. a MCF10A cells were exposed to cortisol (1 μM) and NE (1 μM) for 30 minutes and assessed for DNA damage using the Comet assay. Comet tails indicating DNA strand breaks were visually scored according to intensity (0–4). Representative images shown. b MCF10A cells were exposed to cortisol (1 μM) for 30 minutes and 24 h and mRNA extracted. cDNA was synthesised and amplified in the presence of gene specific primers for NOS2 and β-actin using qRT-PCR. Ct values for NOS2 were normalised against β-actin and fold change calculated using the delta-Ct method. Mean ± SEM is expressed and significance was determined using one-way ANOVA (post hoc Tukey multiple comparisons); *significant increase, *p < 0.05, **p < 0.01, ***p < 0.001. Technical replicate (n = 3). (PPTX 125 kb) [file 13058_2017_823_MOESM2_ESM.pptx]

## Slide 1
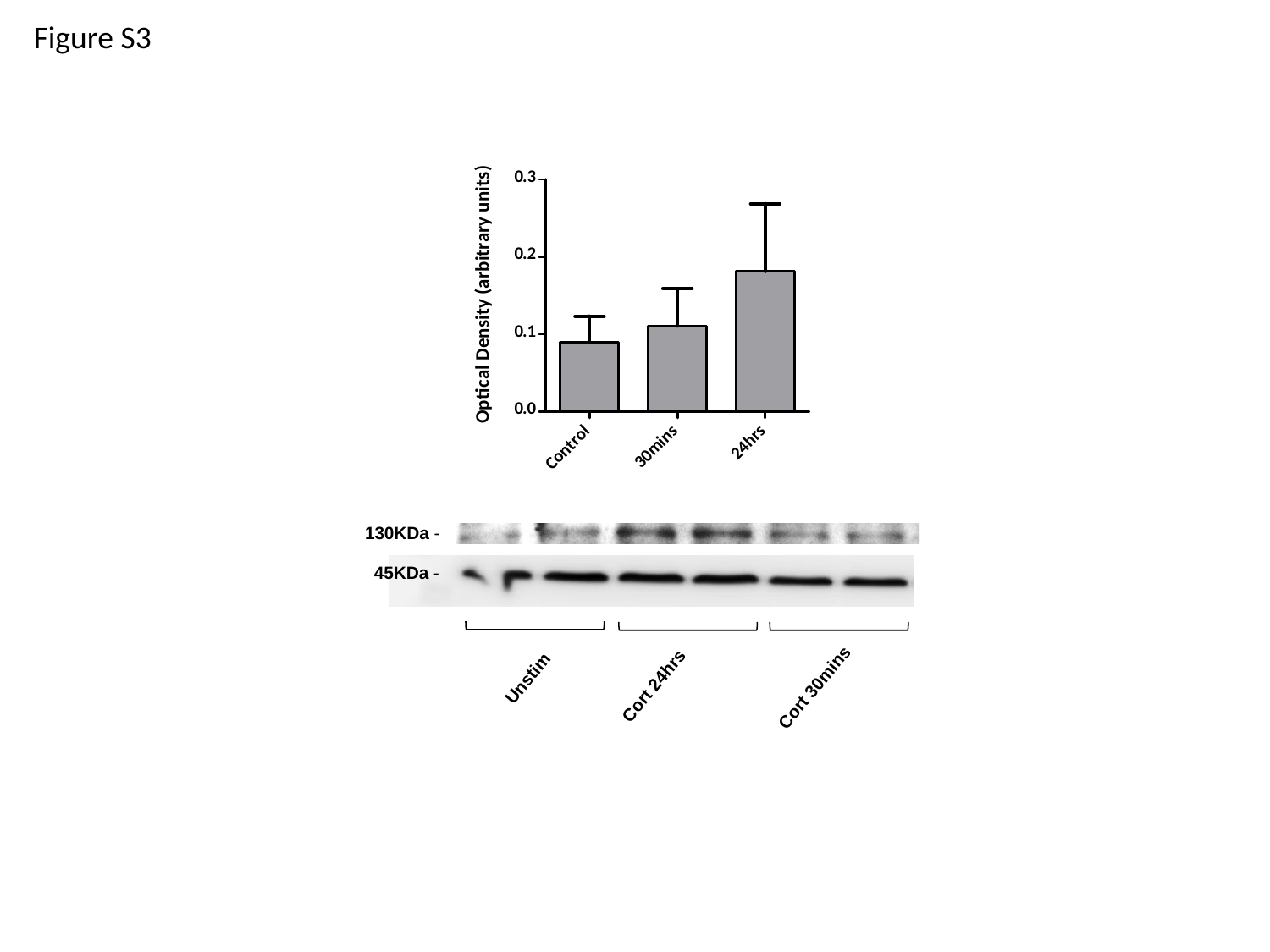

Figure S3
130KDa -
45KDa -
Unstim
Cort 24hrs
Cort 30mins

Supplement: Supplementary file 3 — Expression of iNOS protein is unchanged in response to cortisol. MCF-7 cells were exposed to cortisol (1 μM) for 30 minutes or 24 h. iNOS protein expression was visualised using western blotting. Optical density values were normalised against β-actin. Mean ± SEM is shown. (PPTX 186 kb) [file 13058_2017_823_MOESM3_ESM.pptx]

## Slide 1
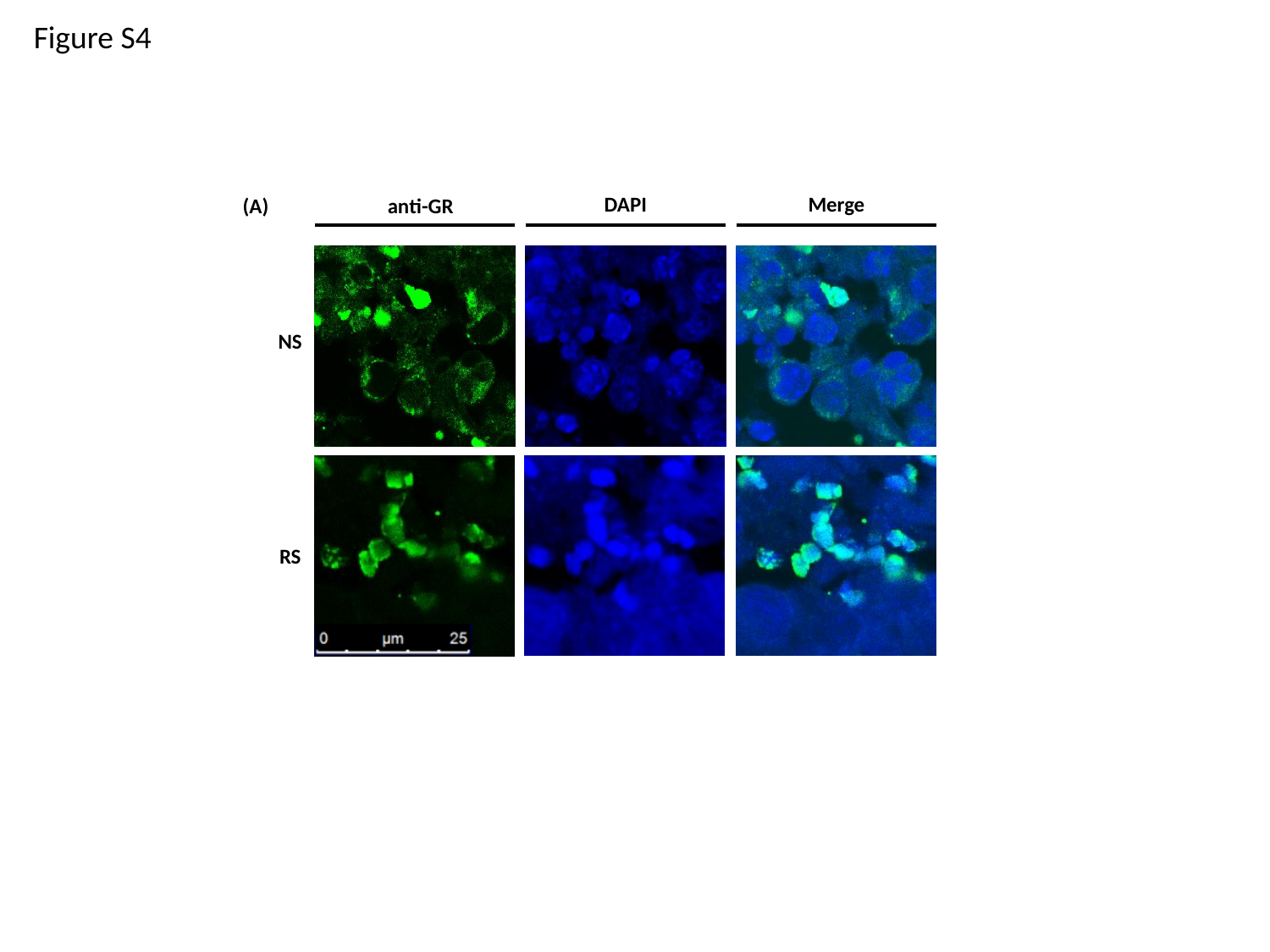

Figure S4
DAPI
Merge
(A)
anti-GR
NS
RS

Supplement: Supplementary file 4 — Glucocorticoid receptor localisation in mice mammary tumours. The 4T1 mouse mammary gland cells were transplanted into the fourth mammary fat pad of female BALB/C mice and the animals randomised into groups either exposed to acute restraint stress or no stress. Tumours were harvested, fixed in paraffin and sectioned subsequent to immunofluorescent detection of glucocorticoid receptor (GR). Representative panels are shown. (PPTX 414 kb) [file 13058_2017_823_MOESM4_ESM.pptx]

## Slide 1
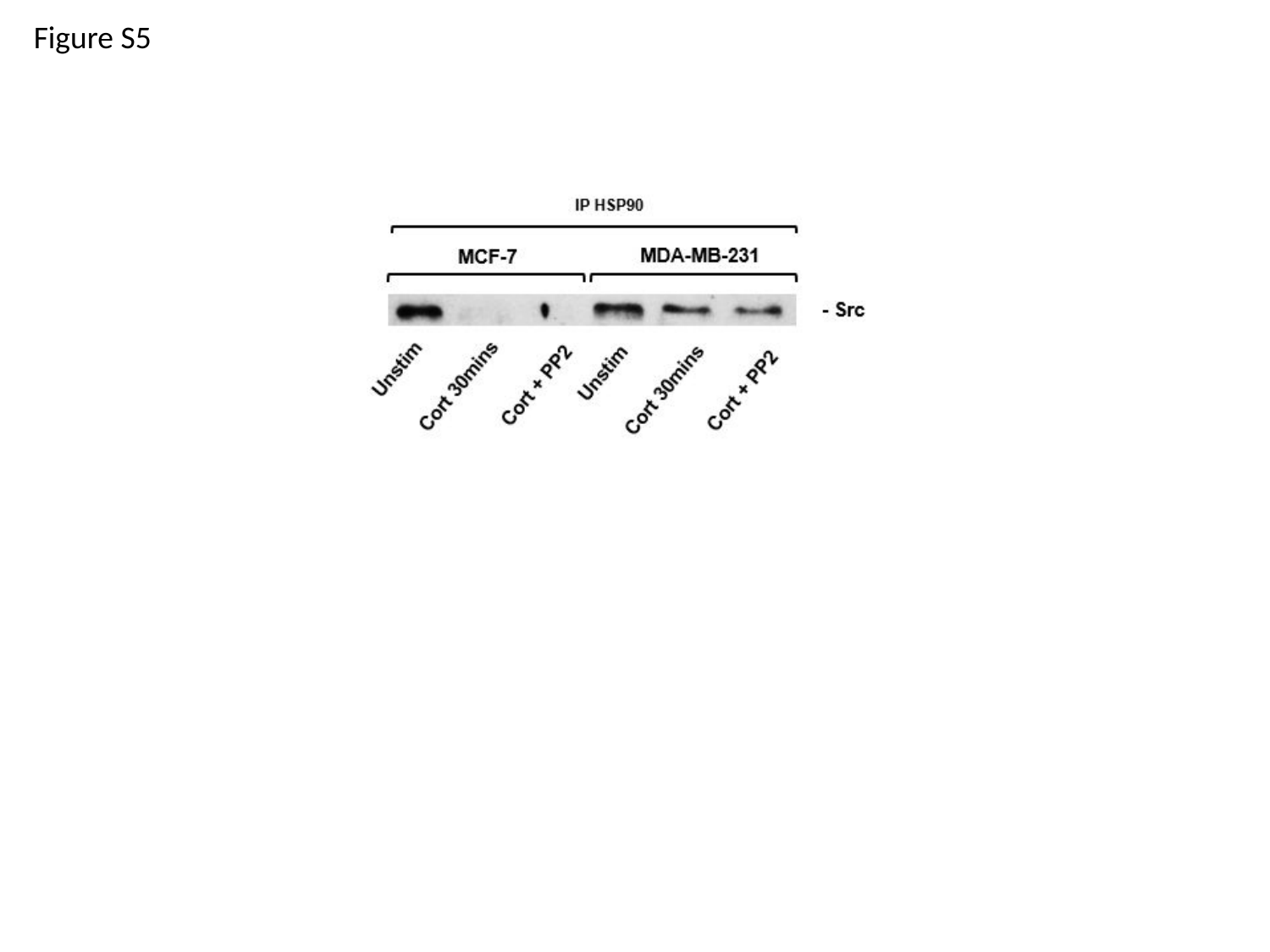

Figure S5

Supplement: Supplementary file 5 — Cortisol induces the dissociation of Src from HSP90. MCF-7 and MDA-MB-231 cells were exposed to cortisol (1 μM) for 30 minutes alongside PP2 (10 μM). Cell lysates were immunoprecipitated for HSP90 and protein levels of Src were visualised using western blotting. (PPTX 52 kb) [file 13058_2017_823_MOESM5_ESM.pptx]
